# Supplementary material for: A transposon expression burst accompanies the activation of Y-chromosome fertility genes during Drosophila spermatogenesis
Source: Nat Commun. 2021 Nov 25;12:6854. doi: 10.1038/s41467-021-27136-4 (PMC8617248; doi:10.1038/s41467-021-27136-4)
Supplement: Supplementary file 1 — Supplementary Information [file 41467_2021_27136_MOESM1_ESM.pdf]

Supplementary Figure 1

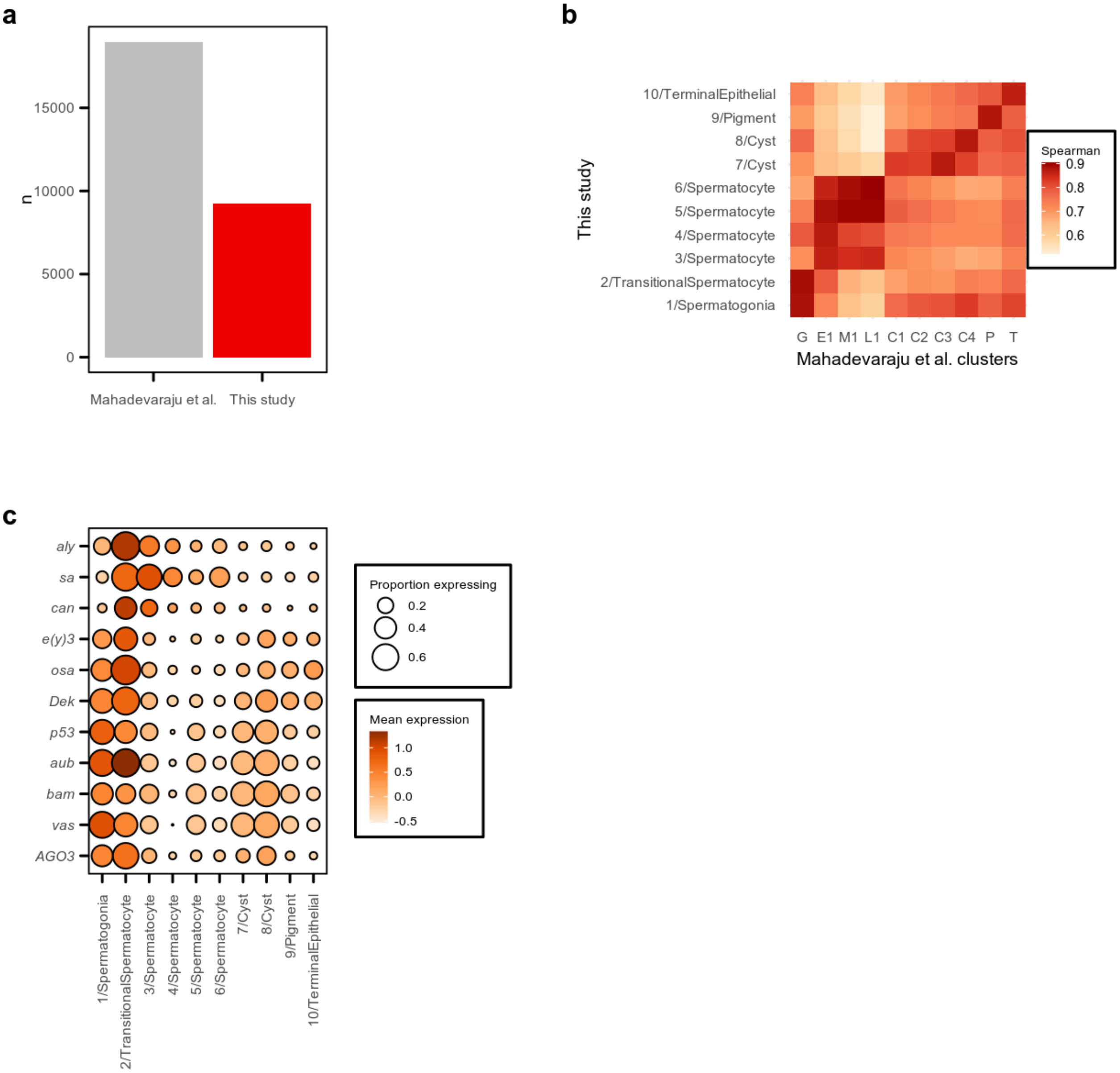

Supplementary Figure 1. scRNA-seq preprocessing and comparison to prior analysis. **a)** Barplot shows the number of cells post-filtering in Mahadevaraju et al. 2021 (gray) and this study (red). **b)** Heatmap shows correlation between scRNA-seq expression estimates derived from our pipeline for all clusters identified in this study compared to clusters identified by Mahadevaraju et al. 2021 (source of data). **c)** Dotplot shows scaled expression of selected spermatocyte and spermatogonia markers. Source data are provided as a Source Data file.

Supplementary Figure 2

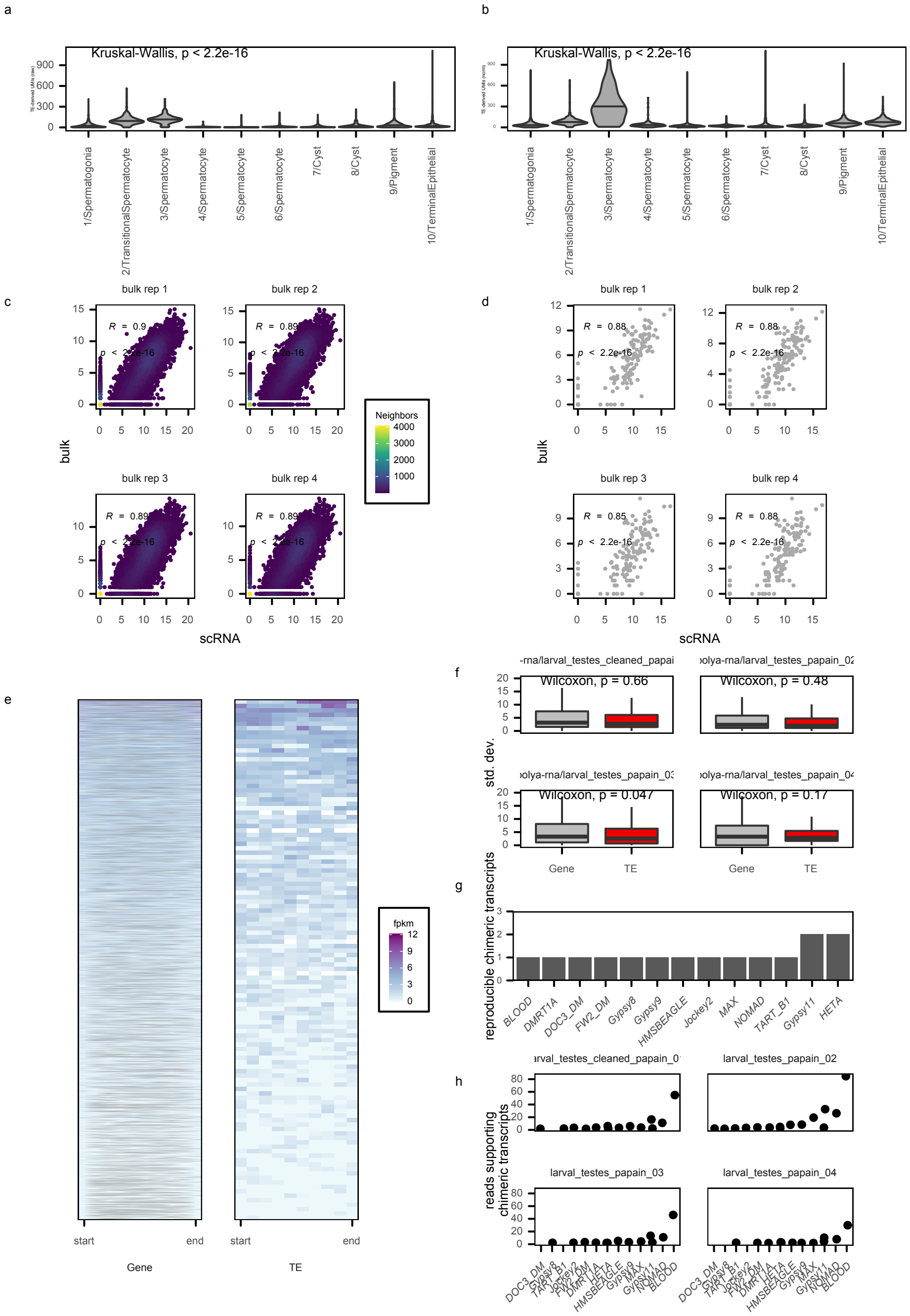

Supplementary Figure 2. TE expression in scRNA-seq data from larval testes. **a)** Violin plots show distributions of raw transposable element-mapping (TE) unique molecular identifier (UMI) counts in each w1118 L3 testis cell cluster. TE counts vary significantly among the clusters (Kruskal-Wallis, one-sided p < 2.2e-16). **b)** Violin plots show distributions of depth normalized TE-mapping UMI counts in each w1118 L3 testis cell cluster. TE counts vary significantly among the clusters (Kruskal-Wallis, one-sided p < 2.2e-16). **c)** Scatterplots show pseudo-bulk expression for both TEs and host genes derived from our w1118 scRNA pipeline (see Methods) versus bulk expression for four w1118 testis poly-A RNA-seq replicates generated by Mahadevaraju et al. 2021 (see Methods). Each replicate shows strong correlation with pseudo-bulk (all Pearson's R >= 0.89, two-sided P<2.2e-16). **d)** Scatterplots show same analysis described in **d** but restricted to TEs (all Pearson's R >= 0.85, two-sided P<2.2e-16). **e)** Heatmaps show distribution of sense-strand bulk poly-A RNA-seq signal for single-isoform host gene mRNAs (left) and detected TEs (right).

Larval bulk poly-A RNA-seq data generated by Mahadevaraju et al. 2021 was mapped to each feature (see Methods) and divided into bins representing one tenth the full length of each feature. **f)** Boxplots show standard deviations of expression across bins for host genes (gray) and TEs (red). Three of four replicates show no significant difference in variability of poly-A signal across bins within features (Wilcoxon rank-sum test P>0.05). Replicate 3 shows a significant difference (Wilcoxon rank-sum test, two-sided P=0.047). For all boxplots, midline represents median, box represents interquartile range (IQR), and whiskers extend >1.5 IQR from the upper or lower quartile. **g)** Bar plot shows number of TE-gene chimeric transcripts reproducibly found in bulk poly-A RNA-seq data, for all TEs with a least one chimeric transcript identified in at least two of the replicate RNA-seq datasets. **h)** For each TE introduced in G, the y-axis position of each point represents the number of uniquely-mapping chimeric reads detected by STAR that support each chimeric transcript (see Methods). Source data are provided as a Source Data file.

Supplementary Figure 3

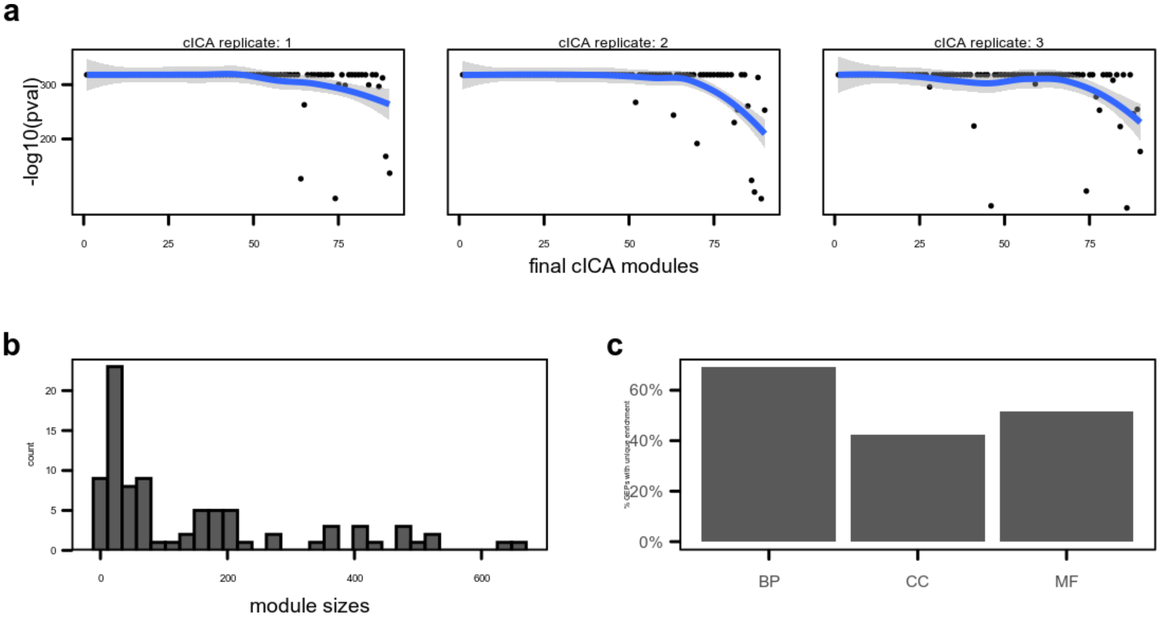

Supplementary Figure 3. Co-expression module detection via consensus approach to Independent Component Analysis (cICA). **a)** Final module gene membership scores were compared to modules identified in replicate consensus independent component analysis (cICA) runs by Spearman correlation. For each module, the two-sided correlation p-values for the best correlated module from each cICA replicate are plotted. Modules (x axis) are ordered by the Spearman correlation with the best match across all three cICA replicates. Error band on the trendline represents fitted value +/- standard error. **b)** Histogram shows distribution of the number of genes or transposable elements (TEs) assigned to the set of modules used in main analysis. **c)** Barplots show percentage of modules uniquely enriched for at least one Biological Process (BP), Cellular Component (CC), or Molecular Function (MF) Gene Ontology Term.Source data are provided as a Source Data file.

Supplementary Figure 4

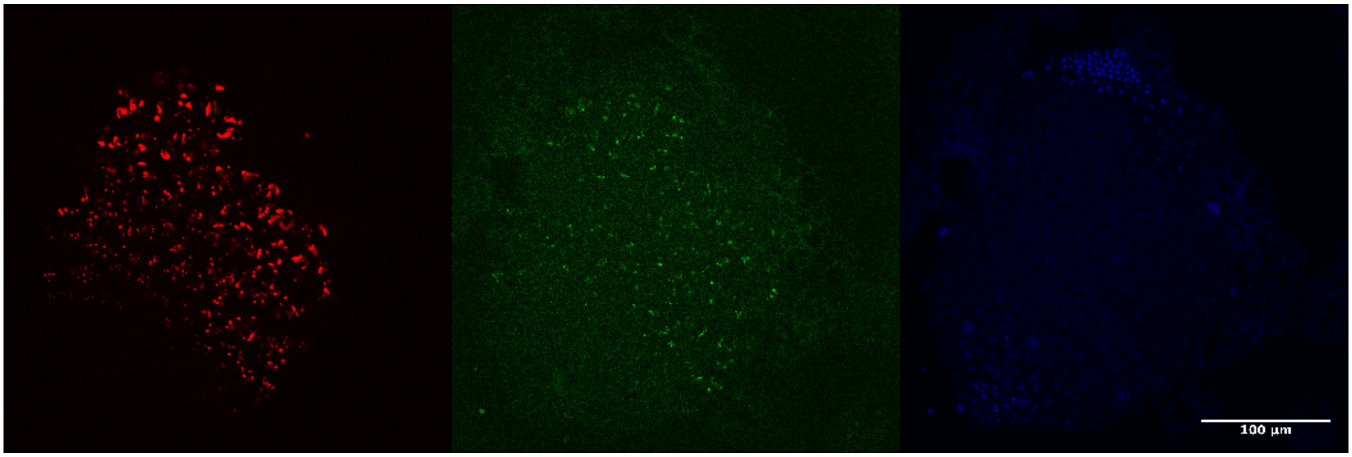

Supplementary Figure 4. ACCORD2 and EAChm expression in L3 testes. Representative slice of multiplexed RNA-FISH in whole-mount 3rd larval instar w1118 testis. Image is split by color channel. *ACCORD2* and *EAChm* expression is detected in the middle region of the testis, where primary spermatocytes are located. Red: *EAChm*; green: *ACCORD2*; blue: DAPI. Brightness and contrast were adjusted separately for each channel.

Supplementary Figure 5

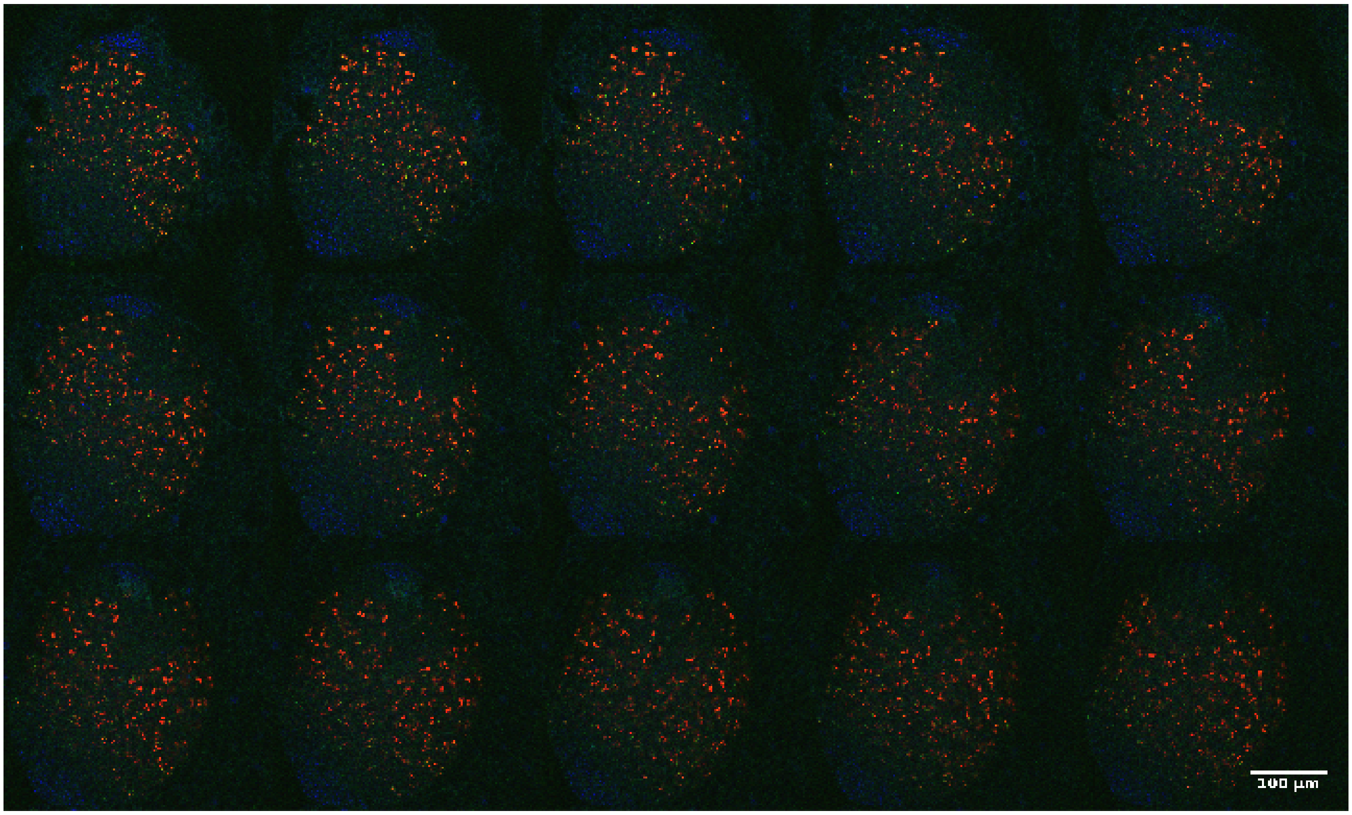

Supplementary Figure 5. ACCORD2 and EAChm expression in L3 testes. Z-slices montage of multiplexed RNA-FISH in whole-mount 3rd larval instar w1118 testis. *ACCORD2* and *EAChm* expression is detected in the middle region of the testis, where primary spermatocytes are located. Red: *EAChm*; green: *ACCORD2*; blue: DAPI. Brightness and contrast were adjusted separately for each channel.

Supplementary Figure 6

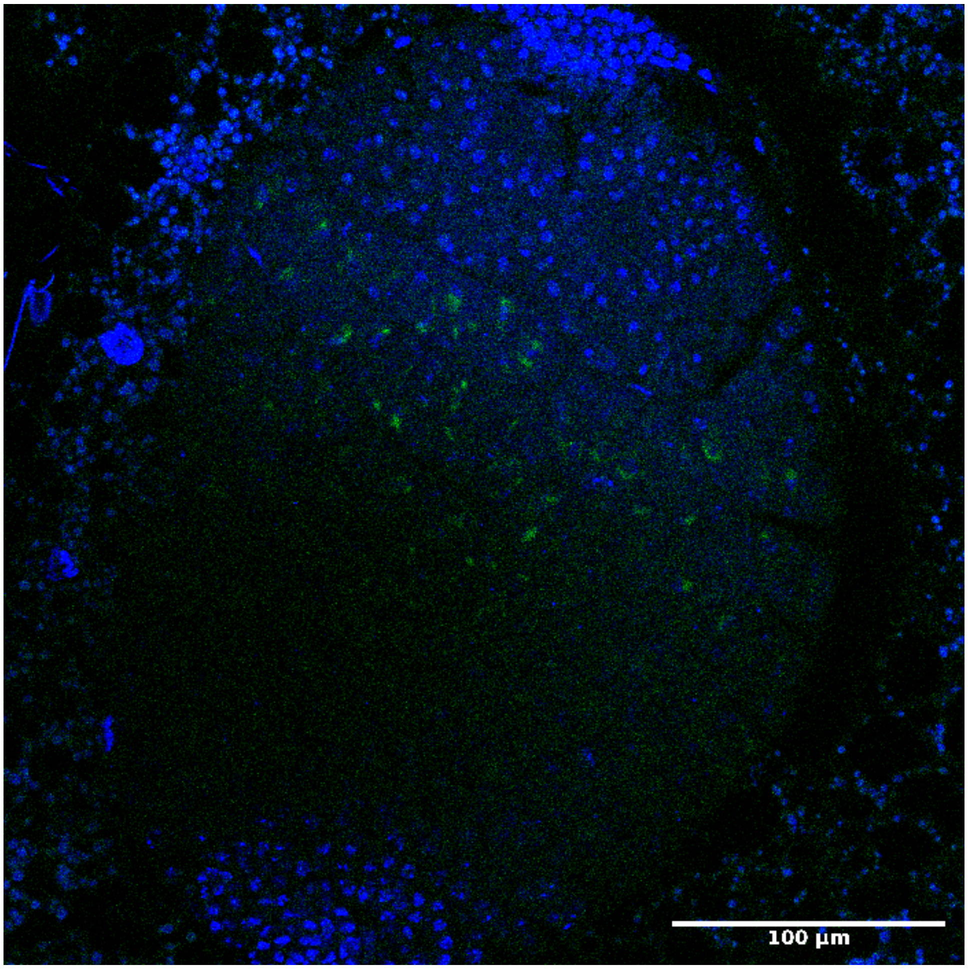

Supplementary Figure 6. QUASIMODO2 expression in L3 testes. Representative RNA-FISH z-slice in whole-mount 3rd larval instar w1118 testis. *QUASIMODO2* expression is detected in the middle region of the testis, where primary spermatocytes are located. Green: *QUASIMODO2*; blue: DAPI. Brightness and contrast were adjusted separately for each channel.

Supplementary Figure 7

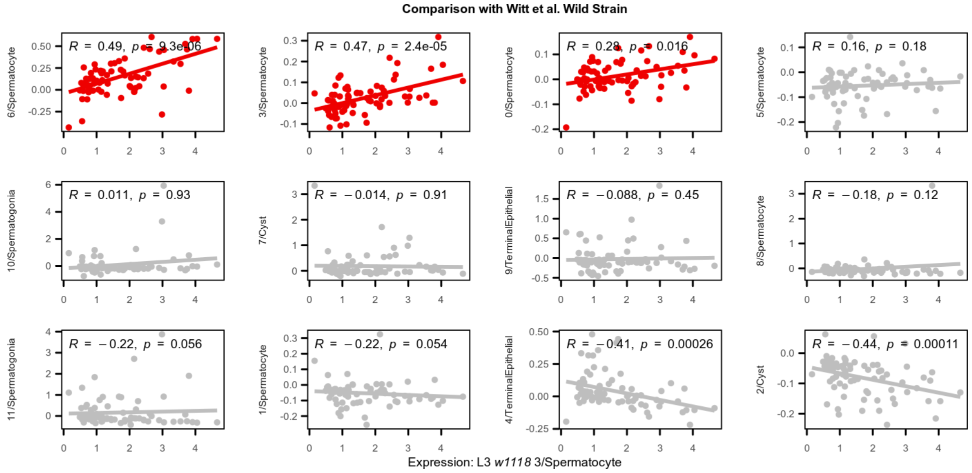

Supplementary Figure 7. TE Expression in adult data. Scatterplots show relationship of transposable element (TE) expression in w1118 Cluster 3/Spermatocytes versus all clusters detected by our pipeline in the "Wild Strain" dataset generated by Witt et al. 2019. Multiple spermatocyte clusters (highlighted in red) show TE expression patterns similar to 3/Spermatocytes. Spearman's correlation with two-sided test was used to compare across datasets. Source data are provided as a Source Data file.

Supplementary Figure 8

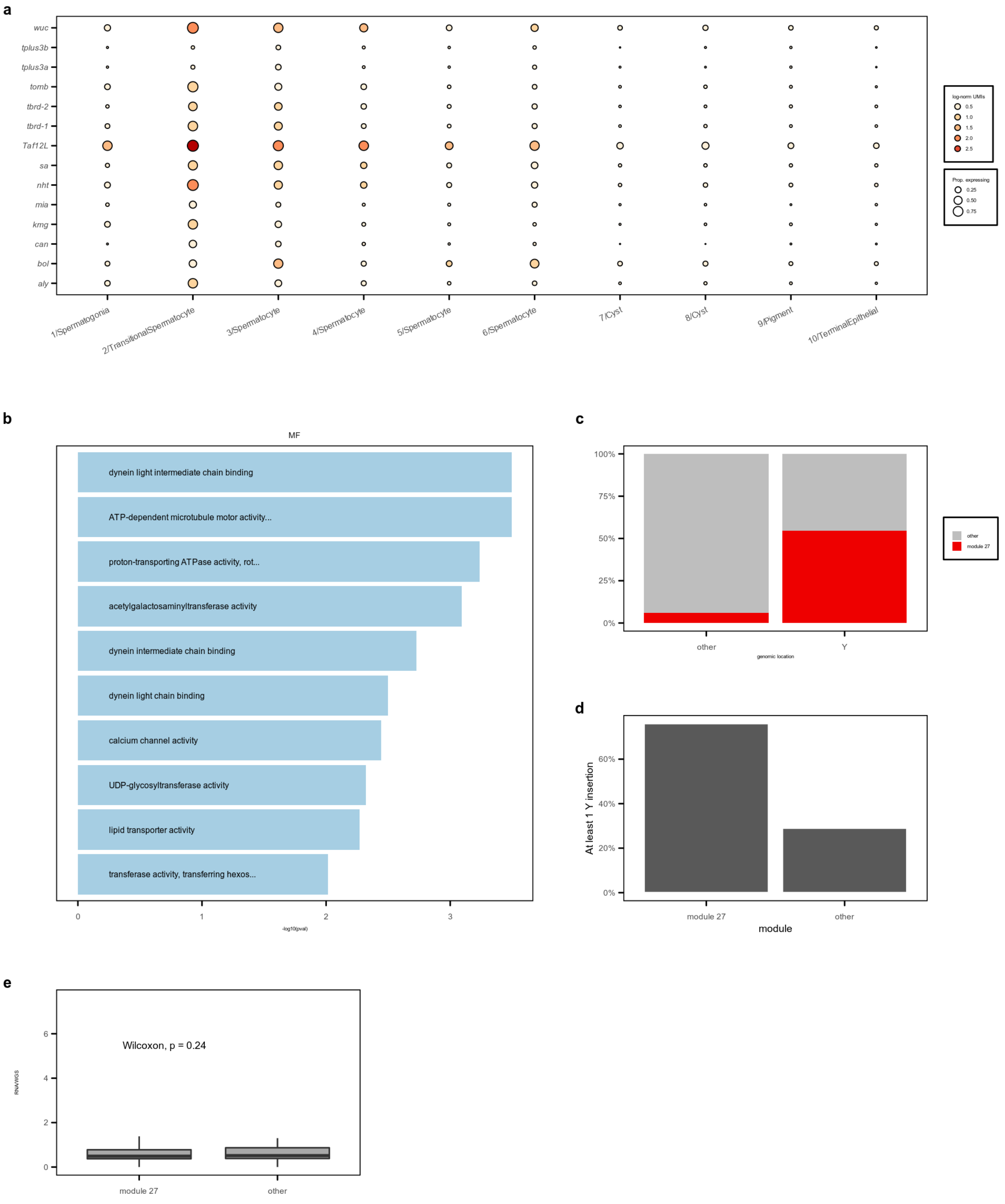

Supplementary Figure 8. Module 27 TEs are enriched on the Y-chromosome. **a)** Dot plot shows expression of selected effectors of spermatocyte transcriptional programs. Color of each dot corresponds to mean normalized and log-transformed expression within cell clusters. Dot size corresponds to the proportion of cells in each cluster expressing the marker. **b)** Bars show strength of top 10 enriched Gene Ontology Molecular Function terms for module 27. **c)** Bar plot shows proportion of Y chromosome genes or other genes used for module detection analysis that are assigned to module 27 (red) or other modules (gray). Fisher's Exact test, two-sided P=1.7e-05. **d)** Barplot shows percentage of transposable elements (TEs) assigned to module 27 or other TEs with at least one Y-linked insertion detected by RepeatMasker in the heterochromatin-enriched assembly described by Chang and Larracuente 2019. **e)** Allele specific analysis of TE expression (see Methods) shows that non-Y-linked copies of module 27 TEs are expressed proportionately to their DNA copy number. N=58 module 27 TEs, 32 other module TEs. For replicates taken together (n=4), Wilcoxon rank-sum test, two-sided P=0.24. For individual replicates, Wilcoxon rank-sum tests

yielded two-sided p-values of 0.5, 0.34, 0.3, and 0.75. For all boxplots, midline represents median, box represents interquartile range (IQR), and whiskers extend >1.5 IQR from the upper or lower quartile. Source data are provided as a Source Data file.

Supplementary Figure 9

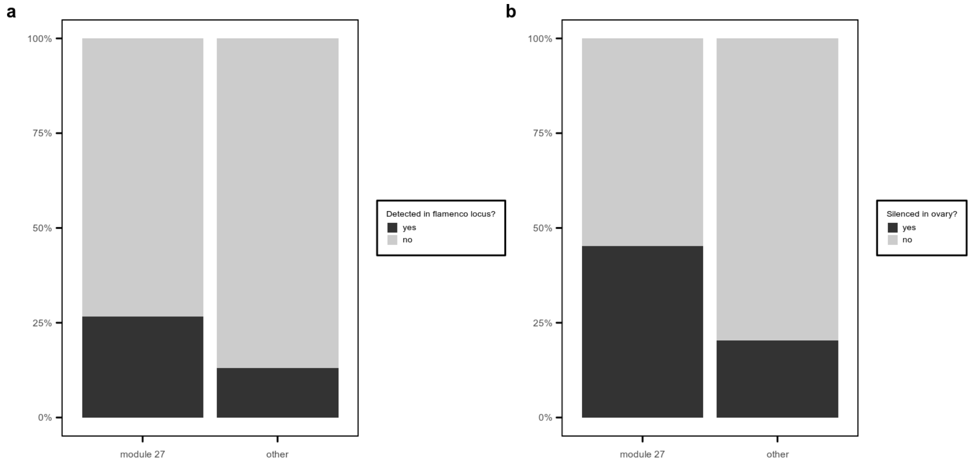

Supplementary Figure 9. Silencing of module 27 TEs in non-spermatocyte contexts. **a)** Barplot shows the percentage of module 27 or other transposable elements (TEs) with fragments in the *flamenco* locus. Module 27 is significantly enriched for TEs represented in the *flamenco* locus (Fisher's Exact Test, two-sided p = 0.03). Dark gray, detected in *flamenco* locus; light gray, not detected. **b)** Barplot shows the percentage of module 27 or other TEs which are silenced by the piRNA pathway in *Drosophila* ovaries. Module 27 is significantly enriched for TEs silenced in ovaries (Fisher's Exact Test, two-sided P=7.6e-4). Dark gray, piRNA pathway regulated in ovary; light gray, piRNA regulaton in ovary not detected. Source data are provided as a Source Data file.

Supplementary Figure 10

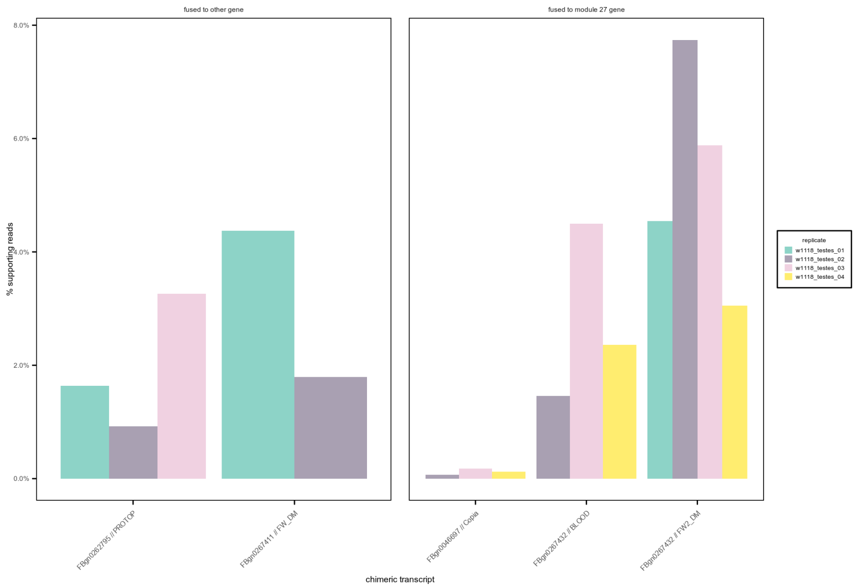

Supplementary Figure 10. Detected fusions between module 27 TEs and Y-linked genes. Fusions between module 27 transposable elements (TEs) and Y-linked genes detected by the Arriba fusion caller in 2 or more w1118 adult testis total RNA-seq replicates. Y-axis shows the percentage of reads that align to the breakpoint that support the fusion call (split reads and discordant pairs). Blue, gray, pink, and yellow correspond to replicates 1-4 respectively. Source data are provided as a Source Data file.

Supplementary Figure 11

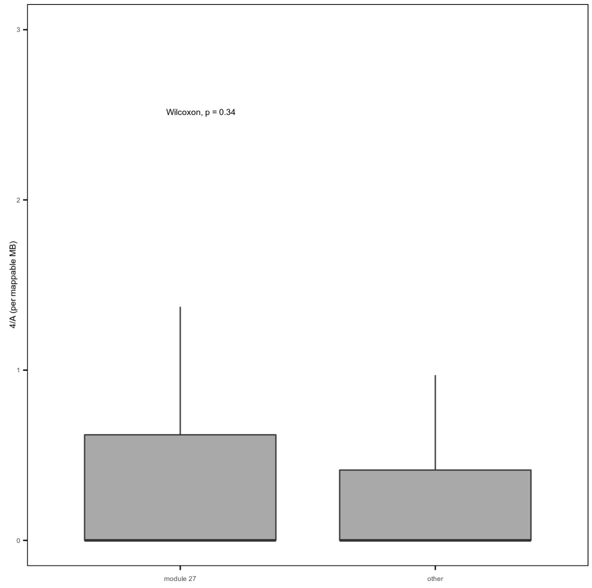

Supplementary Figure 11. Chr4-linked versus autosomal polymorphic insertions. Boxplot showing the ratios of chr4-linked versus autosomal polymorphic insertions per mappable megabase for each transposable element (TE) in the TIDAL-fly database. Module 27 TEs show similar levels of depletion on the 4th chromosome compared to other TEs with polymorphic insertions. N=61 module 27 TEs, 38 other module TEs. Wilcoxon rank-sum test, two-sided P = 0.34. For all boxplots, midline represents median, box represents interquartile range (IQR), and whiskers extend >1.5 IQR from the upper or lower quartile. Source data are provided as a Source Data file.

Supplementary Figure 12

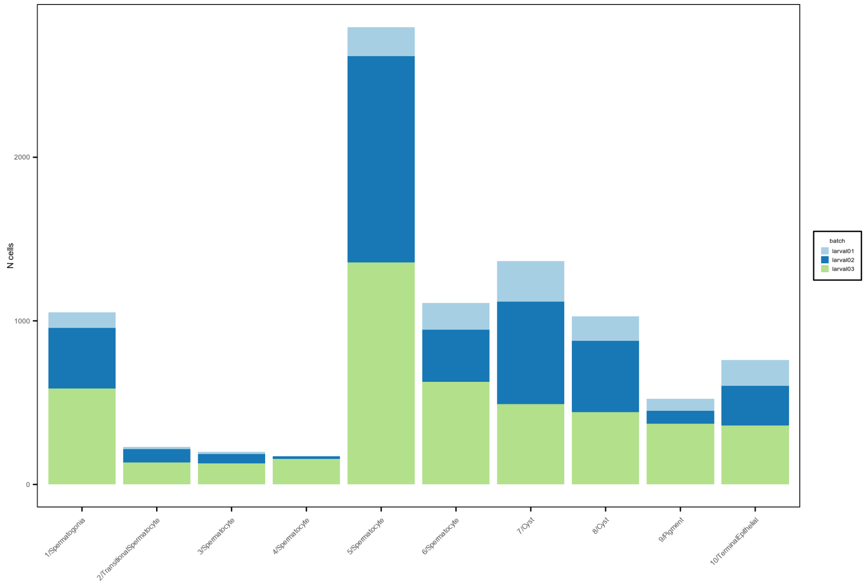

Supplementary Figure 12. Cells in scRNA-seq clusters. Barplot shows the number of cells assigned to each cluster. Light blue, dark blue, and green correspond to single cell RNA-seq replicates 1-3 respectively. Source data are provided as a Source Data file.

Supplementary Figure 13

a **b**

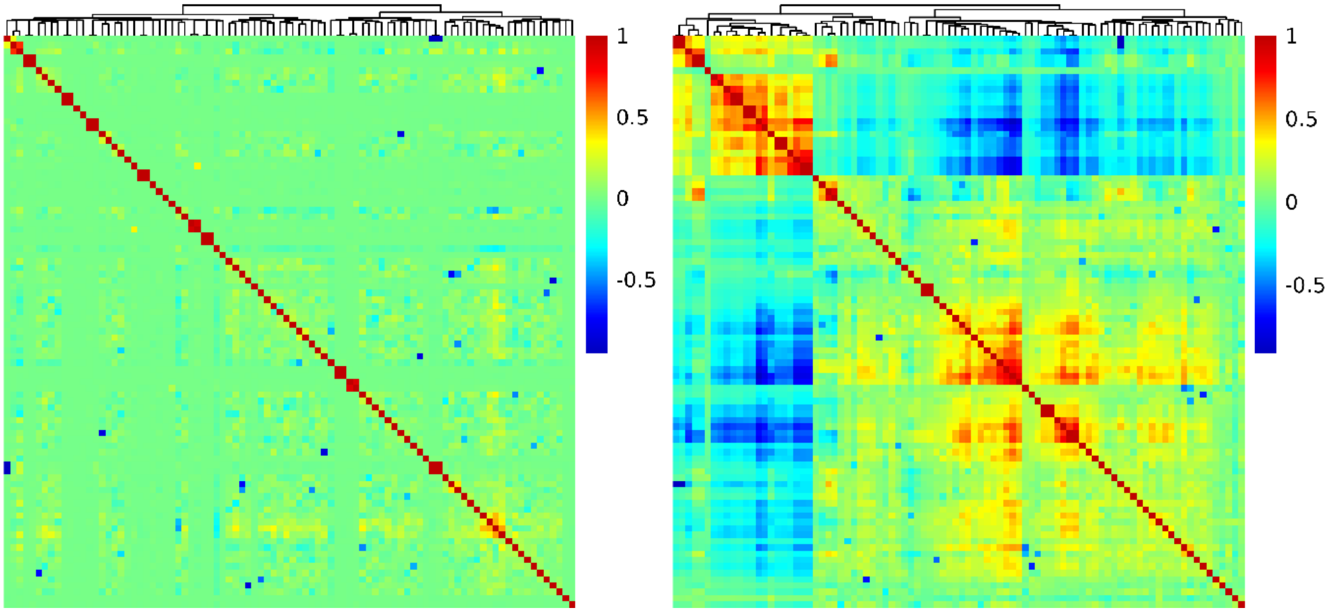

Supplementary Figure 13. Redundancy and TE content of modules. **a)** Clustering of modules by cell usage score (consensus independent component analysis (ICA) source matrix). **b)** Clustering of modules by gene membership score (consensus ICA mixing matrix). For A and B `1-Pearson's R` is used as a distance metric. Source data are provided as a Source Data file.
