# Supplementary material for: A transposon expression burst accompanies the activation of Y-chromosome fertility genes during Drosophila spermatogenesis
Source: Nat Commun. 2021 Nov 25;12:6854. doi: 10.1038/s41467-021-27136-4 (PMC8617248; doi:10.1038/s41467-021-27136-4)
Supplement: Supplementary file 3 — Description of Additional Supplementary Files [file 41467_2021_27136_MOESM3_ESM.pdf]

Description of Additional Supplementary Files

File name: Supplementary Data 1

Description: List of module 27 TEs. TEs significantly associated with module 27 are listed along with their module membership score.

File name: Supplementary Data 2

Description: List of module 27 genes. Genes significantly associated with module 27 are listed along with their module membership score.

File name: Supplementary Data 3

Description: Differentially expressed genes from the scRNA-seq larval testes dataset. Differentially expressed genes for each cluster compared to the union of all other clusters, as identified by scanpy are listed in this table. Two-sided tests of significance were performed and both unadjusted and adjusted p values are provided.

File name: Supplementary Data 4

Description: RNAFISH probes. Target and probe sequences used for RNA-FISH along with design parameters.
