# Supplementary material for: A transposon expression burst accompanies the activation of Y-chromosome fertility genes during Drosophila spermatogenesis
Source: Nat Commun. 2021 Nov 25;12:6854. doi: 10.1038/s41467-021-27136-4 (PMC8617248; doi:10.1038/s41467-021-27136-4)
Supplement: Supplementary file 8 — Reporting Summary [file 41467_2021_27136_MOESM8_ESM.pdf]

nature portfolio

Corresponding author(s): Christopher E. Ellison

Last updated by author(s): Oct 12, 2021

Reporting Summary

Nature Portfolio wishes to improve the reproducibility of the work that we publish. This form provides structure for consistency and transparency in reporting. For further information on Nature Portfolio policies, see our Editorial Policies and the Editorial Policy Checklist.

Statistics

For all statistical analyses, confirm that the following items are present in the figure legend, table legend, main text, or Methods section.

n/a	Confirmed	
☐	☒	The exact sample size (*n*) for each experimental group/condition, given as a discrete number and unit of measurement
☐	☒	A statement on whether measurements were taken from distinct samples or whether the same sample was measured repeatedly
☐	☒	The statistical test(s) used AND whether they are one- or two-sided<br>*Only common tests should be described solely by name; describe more complex techniques in the Methods section.*
☐	☒	A description of all covariates tested
☒	☐	A description of any assumptions or corrections, such as tests of normality and adjustment for multiple comparisons
☐	☒	A full description of the statistical parameters including central tendency (e.g. means) or other basic estimates (e.g. regression coefficient) AND variation (e.g. standard deviation) or associated estimates of uncertainty (e.g. confidence intervals)
☐	☒	For null hypothesis testing, the test statistic (e.g. *F*, *t*, *r*) with confidence intervals, effect sizes, degrees of freedom and *P* value noted<br>*Give P values as exact values whenever suitable.*
☒	☐	For Bayesian analysis, information on the choice of priors and Markov chain Monte Carlo settings
☒	☐	For hierarchical and complex designs, identification of the appropriate level for tests and full reporting of outcomes
☐	☒	Estimates of effect sizes (e.g. Cohen's *d*, Pearson's *r*), indicating how they were calculated

Our web collection on statistics for biologists contains articles on many of the points above.

Software and code

Policy information about availability of computer code

Data collection	The wget (v1.14) command line utility was used to download data from public repositories.
Data analysis	Custom code (with README containing full description) available at https://github.com/Ellison-Lab/TestisTEs2021<br>Publicly available software used for this study is listed below:<br>•STAR v2.7.3/5/9•Cellranger v3.1.0•Snakemake v6.1.0•Mamba v0.9.1•Fastp v0.20.0•Cutadaptv3.2.0•RepeatMasker•Scrublet v0.2.1•Scanpy v1.6.0•Garnett v0.2.17•Fdrtool•Scikit-learn•Deeptools v3.3.1•Picardtools v2.22.1•Samtools v1.10.0•Mosdepth v0.3.1•Rsamtools v2.0.0•BWA-MEM2 v2.0•Arriba v2.1.0•R v4.0.3•Tidyverse v1.3.0•Ragg v1.1.2•Ggtext v0.1.1•Arrow v3.0.0•Jsonlite v1.7.2•Readxl v1.3.1•Rtracklayer v1.50.0•GenomicRanges v1.42.0•Patchwork v1.1.1•Png v0.1-7•Grid v4.0.3•Magick v2.7.1•Extrafont v0.17•Ggforce v0.3.3•Ggrepel v0.9.1•Genomicfeatures v1.42.3•Ggpubr v0.4.0•Ggpointdensity v0.1.0•Complexheatmap v2.6.2•Broom v0.7.5•VariantAnnotation v1.36.0•BLAST v2.11.0

For manuscripts utilizing custom algorithms or software that are central to the research but not yet described in published literature, software must be made available to editors and reviewers. We strongly encourage code deposition in a community repository (e.g. GitHub). See the Nature Portfolio guidelines for submitting code & software for further information.

Data

Policy information about availability of data

All manuscripts must include a data availability statement. This statement should provide the following information, where applicable:

- Accession codes, unique identifiers, or web links for publicly available datasets
- A description of any restrictions on data availability
- For clinical datasets or third party data, please ensure that the statement adheres to our policy

The w1118 whole genome sequencing data and w1118 adult testes bulk total RNA-seq data generated in this study have been deposited in the National Center for Biotechnology Information Sequence Read Archive (NCBI SRA) under accession code PRJNA727858 [https://www.ncbi.nlm.nih.gov/bioproject/PRJNA727858/].

Previously published sequencing data used in this study is available at the NCBI SRA via project accessions PRJNA475132 [https://www.ncbi.nlm.nih.gov/bioproject/475132], PRJNA548742 [https://www.ncbi.nlm.nih.gov/bioproject/PRJNA548742], PRJNA518743 [https://www.ncbi.nlm.nih.gov/bioproject/PRJNA518743], and PRJNA197267 [https://www.ncbi.nlm.nih.gov/bioproject/PRJNA197267].

Source data are provided with this paper via zenodo [https://doi.org/10.5281/zenodo.5554937]

Field-specific reporting

Please select the one below that is the best fit for your research. If you are not sure, read the appropriate sections before making your selection.

☒ Life sciences ☐ Behavioural & social sciences ☐ Ecological, evolutionary & environmental sciences

For a reference copy of the document with all sections, see nature.com/documents/nr-reporting-summary-flat.pdf

Life sciences study design

All studies must disclose on these points even when the disclosure is negative.

Sample size	No sample size calculations were performed. Sample sizes were chosen to be sufficiently large while maintaining the ability to practically perform the assays. All sample sizes meet or exceed common standards used in this field.
Data exclusions	No sample-level data exclusion was performed. Standard filtering of single-cell RNA-seq data was performed to exclude putative doublets and other artifacts.
Replication	All experiments include at least two replicates. All attempts to replicate the results were successful.
Randomization	There was no allocation of subjects into groups, so randomization was not performed.
Blinding	Blinding was not used because there are no case/control groups: the data are primarily NGS data analyzed by an automated set of scripts.

Reporting for specific materials, systems and methods

We require information from authors about some types of materials, experimental systems and methods used in many studies. Here, indicate whether each material, system or method listed is relevant to your study. If you are not sure if a list item applies to your research, read the appropriate section before selecting a response.

Materials & experimental systems

n/a	Involved in the study	
☒	☐	Antibodies
☒	☐	Eukaryotic cell lines
☒	☐	Palaeontology and archaeology
☐	☒	Animals and other organisms
☒	☐	Human research participants
☒	☐	Clinical data
☒	☐	Dual use research of concern

Methods

n/a	Involved in the study	
☒	☐	ChIP-seq
☒	☐	Flow cytometry
☒	☐	MRI-based neuroimaging

Animals and other organisms

Policy information about studies involving animals; ARRIVE guidelines recommended for reporting animal research

Laboratory animals	Drosophila melanogaster strain w1118 was exclusively used for this study. Where noted in methods, L3 male larvae, 3-5 day male adults, or 3-5 day female adults were used.

Wild animals	The study did not involve wild animals
Field-collected samples	The study did not involve field-collected samples
Ethics oversight	No special ethical considerations or constraints are required for the Drosophila melanogaster model organism. Standard husbandry practices were applied during the generation of all data for this study.

Note that full information on the approval of the study protocol must also be provided in the manuscript.
